# The Treatment of Teeth during Pregnancy

**Published:** 1892-03

**Authors:** J. W. Van Doorn

**Affiliations:** Cleveland, O.


### The Treatment of the Teeth During Pregnancy.

BY DR. J. W. VAN DOORN, CLEVELAND, O.

I cannot hope in what I may say on this subject, to add anything new, or to give anything in the way of data, from cases under my own observation.

I was induced to choose the subject for two reasons: first, that it seemed likely to provoke ample discussion because of the many opportunities for differences of opinion which present; secondly, that by the discussion, ideas, new to the younger men of the Society, at least would perhaps be brought out.

For no man can have practiced successfully, for many years, without having had some cases of this character under his care. What he did for them, outside of the actual operations performed, doubtless he will remember, together with the results, gratifying or otherwise. Let each one then contribute his experi-

Discussions reported for The Ohio Journal, by W. H. McKerrall, D.D.S. Read before the Cleveland Dental Society, December, 1891.

ence and his opinions derived from that experience, and we shall get at the matter thoroughly and beneficially.

The dentist no longer confines his attention to the teeth only. He looks beyond. This is not so much a matter of choice as it is a matter of necessity. We do not seek out the conditions, but rather are confronted by them. And so it follows that we find our duty and our sphere widening in many directions, one of which is suggested by our subject this evening.

Those of you who already measure your experience by years, can, without doubt, recall instances where the patient who came to you had had little or no dental work done, until called upon to fulfill the duties of maternity. You can probably also recall instances in which the work you have done, beautiful and stable for years, suddenly and under like conditions, becomes anything but a protection to the teeth, a sorry exhibition of your skill, a disappointment to the patient and a matter of more or less vexation to yourself, blameless though you know yourself to be.

For it is not always a patient who is able or willing to discriminate.

The fact is therefore apparent that during the period of gestation the teeth are likely to become sensitive and carious; and that we, in treating them, need to exercise judgment as well as skill. For our efforts, if successful, benefit not the mother alone but find perhaps their greatest usefulness in the developing embryo.

What actually takes place? On this point authorities differ.

Is the decay of teeth during this crucial period, due to the absolute abstraction of the chemical constituents; is it due to what Dr. Abbott calls a "melting away" of the salts of their bodily transfer through the circulatory system to the embryo in the uterus?

Or is it due to the action of the acid secretions now constantly present in the mouth?

This much we know. That the teeth become painful and sensative to an exalted degree. Undoubtedly this is due to the action of acids. For it is no unusual thing for the teeth to be thus affected among the perfectly well. Now comes another phase. The mother often exhibits an abnormal craving for lime, chalk, slate pencils and the like.

Some say that this appetite betokens Nature's effort to supply the waste. Others that it is simply Nature's attempt to afford an alterative for the general acid condition of the system which now obtains.

"If to supply the waste," say the first, "why the craving for lime *as lime*, which is unassimilable? Why the craving for earthy phosphates which, *as earthy phosphates*, chemistry proves useless to the animal economy." This would seem to support the theory that the system simply needs an alkali, and, having that, is satisfied. This is not to build up, not to make good a deficiency, but merely to correct an acid condition and possibly arrest destructive metamorphosis.

On the other hand, what do we find corroborative of the theory of waste and the ability to repair that waste by the introduction of soluble salts of lime? One of the first things I found was this assertion of Prof. Black's, anything but corroborative you will agree with me. He says: "A tooth once soft, always soft; no change takes place in that tooth, because of the introduction of lime salts into the system." Questions leap to our minds that we would like to ask Dr. Black, if this be so; but as we look the field over we find him hopelessly in the minority, and so decide rather to select opinions from the other side than to devote any time to argument on this point.

Dr. Dwinelle says, in effect, "Keep the system well supplied with the bone phosphate of lime, diet always toward the alkaline, use locally ant-acid mouth washes and dentifrices."

This is a summary of his treatment, and I transcribe one case of many cited by him, as proof of its efficiency. The proof, it is true, is with reference more especially to the child than to the mother, but that is hardly an impairment of the theory, I think, since the two are scarcely to be considered separately during this period.

"Mrs. C. had passed through two painful periods of gestation, with excessive acid secretions and vomiting most of the time, giving birth to a girl ten years ago, and two years later to a boy.

The girl's first teeth were remarkably poor. All that were erupted of the second set can be cut away as though they were were chalk. The teeth of the boy are all even worse than those of the sister.

"When the mother became pregnant a third time, with all the symptoms in an unusually aggravated form, I at once put her on the phosphate treatment and prescribed diet together with general tonics. She at once began to mend. The acid secretions and eructations abated entirely; her vomiting ceased at the end of the second month, and did not return. She passed through a remarkably healthy gestation and gave birth to a boy. You may anticipate that I watched the eruption of his first teeth, with interest. They proved to be of the finest quality. The six-year teeth are already fully erupted, perfect in all respects, and of unusual density."

I could have wished that Dr. Dwinelle had given us some statements with reference to the condition and character of the mother's teeth originally. That would perhaps have given more light on the result in the first two children, without detracting any from the value of the treatment in the last case. Something, too, in the way of a statement as to the time of eruption of the teeth in the last case, would have been a help to us, provided it was either early or late.

Dr. Darby, commenting on the remark of Prof. Black's, already quoted, says, "I differ very materially from Dr. Black, on this point, because I am positive that I have seen changes take place in the teeth for the better; teeth that were soft, in early life, becoming hard and good later in life. I have also seen in my own practice, cases where a diet composed largely of phosphates was introduced, where the diet had been of a different character, containing little phosphates, and the teeth being in bad condition; and the favorable results upon the teeth, following the change in diet, were truly wonderful." Dr. Darby then goes on to say, "I think, if we could introduce into the system of women, during the period of gestation, a greater proportion of the phosphates in their food, there would be a great improvement in the teeth of their children."

And so we might continue adducing experiences and opinions. The concensus of the best writers seems to be, summarily speaking, that the system should be kept well nourished during this period, particularly with appropriate salts of lime.

Now let us look at the matter more in detail. First as to ordinary diet. Let the mother eat sufficiently of bread made from coarse flour, in which has been retained the phosphate-containing outer-capsule of the wheat kernel. I suppose Graham bread, rye bread, and particularly corn bread, would recommend themselves in this respect.

Corn-meal mush, oat meal and rolled wheat, of course, would figure prominently as desirable foods.

Plenty of well cooked beef, baked potatoes and soups made of such vegetables as peas, beans and so forth, would have their place.

Unfortunately, however, the taste may reject many of these simple and more natural means of affording a supply of the phosphates. It then becomes necessary to furnish them in another form, readily assimilable and palatable.

From among the variety of ways in which this can be done, I choose, as at once the simplest and most effectual, the Syrup of the Lacto-phosphate of Lime, to be given in doses of a dessert-spoonful in a little water after meals. This is simple, agreeable to the taste and very effective, if we may rely on the testimony of those who have used it.

Lactic acid to be given in alternate weeks. Dose: A table-spoonful, to which has been added gr. xii. Caleii Phosphatis (freshly prepared), three times a day, is another form in which we may administer the phosphate.

So much for the direct treatment. Incidentally we may be required to alleviate the frequently occurring nausea of this period. For this, Ingluvin, in doses of gtt. v. *ad.* x., as needed, is recommended.

Tonics, as indicated, should be used, and of course with them the proper means to keep the movement of the bowels regular.

In closing this part of the subject, the following questions suggest themselves:

What causes the acid conditions at this time more than another? Is it due to mal-nutrition, mal-assimilation? When nutrition is good, is the acid condition as marked? If it is, how do you account for it? Why do the teeth of the mother soften? Is it because of the dissolving influence of the acid secretions? Is it because the teeth directly go to supply the embryo, or because they are robbed of their quota of nourishment by the occupant of the womb?

In supplying the mother's system with an abundance of bone-making foods, is or is there not danger of excessive or premature ossification of the embryo, making labor difficult?

As briefly as possible, let us now consider the subject of operations at this time. Dr. Darby says, "My own practice has been, during the period of pregnancy, to fill the teeth with plastics only, as much as possible avoiding large operations and the use of metallic substances." Such is practically the unanimous opinion of the profession. The following rules are suggested:

No extracting, if it can be avoided.

No administering of gas under any circumstances.

In filling teeth, plastics only, gutta-percha preferred to the cements, as the latter rapidly succumb to the acidity of the oral secretions.

That anything fatal must perforce come of an infringement of these rules is not the point sought to be established. Direful results are possible, if not probable. It is not enough for me that you say, "Why, I have given gas to hundreds of women in that condition with no bad results;" or, "I have extracted lots of teeth for pregnant women without harm!" As I look at it, this is not a legitimate field for experimentation, to discover how much we can do, but rather a field for marked conservativeness of action.

I now leave the subject, feeling, in the words of a familiar quotation, somewhat perverted, that "I may have said the things I ought not to have said, and may not have said the things which I ought to have said." If the latter part of this be true, there remains the consoling thought that they will be said and better said by those who follow me.

